# Genome-Scale Investigation of the Metabolic Determinants Generating Bacterial Fastidious Growth

**DOI:** 10.1128/mSystems.00698-19

**Published:** 2020-03-31

**Authors:** Léo Gerlin, Ludovic Cottret, Sophie Cesbron, Géraldine Taghouti, Marie-Agnès Jacques, Stéphane Genin, Caroline Baroukh

**Affiliations:** a LIPM, Université de Toulouse, INRAE, CNRS, Castanet-Tolosan, France; b IRHS, INRAE, AGROCAMPUS-Ouest, Université d’Angers, Beaucouzé, France; Chan Zuckerberg Biohub

**Keywords:** metabolic network, metabolic pathways, metabolic modeling, robustness, pathogen, growth, *Xylella fastidiosa*, *Xanthomonas*, fastidious

## Abstract

High proliferation rate and robustness are vital characteristics of bacterial pathogens that successfully colonize their hosts. The observation of drastically slow growth in some pathogens is thus paradoxical and remains unexplained. In this study, we sought to understand the slow (fastidious) growth of the plant pathogen Xylella fastidiosa. Using genome-scale metabolic network reconstruction, modeling, and experimental validation, we explored its metabolic capabilities. Despite genome reduction and slow growth, the pathogen’s metabolic network is complete but strikingly minimalist and lacking in robustness. Most alternative reactions were missing, especially those favoring fast growth, and were replaced by less efficient paths. We also found that the production of some virulence factors imposes a heavy burden on growth. Interestingly, some specific determinants of fastidious growth were also found in other slow-growing pathogens, enriching the view that these metabolic peculiarities are a pathogenicity strategy to remain at a low population level.

**IMPORTANCE** Xylella fastidiosa is one of the most important threats to plant health worldwide, causing disease in the Americas on a range of agricultural crops and trees, and recently associated with a critical epidemic affecting olive trees in Europe. A main challenge for the detection of the pathogen and the development of physiological studies is its fastidious growth, as the generation time can vary from 10 to 100 h for some strains. This physiological peculiarity is shared with several human pathogens and is poorly understood. We performed an analysis of the metabolic capabilities of X. fastidiosa through a genome-scale metabolic model of the bacterium. This model was reconstructed and manually curated using experiments and bibliographical evidence. Our study revealed that fastidious growth most probably results from different metabolic specificities such as the absence of highly efficient enzymes or a global inefficiency in virulence factor production. These results support the idea that the fragility of the metabolic network may have been shaped during evolution to lead to the self-limiting behavior of X. fastidiosa.

## Abstract (precis)

Xylella fastidiosa is one of the most important threats to plant health worldwide, causing disease in the Americas on a range of agricultural crops and trees, and recently associated with a critical epidemic affecting olive trees in Europe. A main challenge for the detection of the pathogen and the development of physiological studies is its fastidious growth, as the generation time can vary from 10 to 100 h for some strains. This physiological peculiarity is shared with several human pathogens and is poorly understood. We performed an analysis of the metabolic capabilities of X. fastidiosa through a genome-scale metabolic model of the bacterium. This model was reconstructed and manually curated using experiments and bibliographical evidence. Our study revealed that fastidious growth most probably results from different metabolic specificities such as the absence of highly efficient enzymes or a global inefficiency in virulence factor production. These results support the idea that the fragility of the metabolic network may have been shaped during evolution to lead to the self-limiting behavior of X. fastidiosa.

## INTRODUCTION

Optimal growth rate and robustness against environmental perturbations are major properties contributing to bacterial fitness (1–3). For example, experimental evolution of the plant pathogen Ralstonia solanacearum on several hosts revealed that bacteria acquired both enhanced growth rate and better robustness through enlarged catabolic capacities, showing that these phenotypes are under selective pressure (4, 5). The robustness of virulence functions in this pathogen is also illustrated by the plethora of effectors transiting through the type 3 secretion system, as well as for the regulatory and metabolic networks (6, 7). These traits were shown to be crucial to invade the host and bypass its immune system (7, 8). However, it is also well documented that some bacterial pathogens are slow growing, qualified as fastidious, such as *Legionella* species (9), *Brucella* species (9), and Bordetella pertussis (10–12). Fastidiousness refers to an arduous laboratory handling, and these difficulties were first hypothesized as a consequence of a lack of knowledge of nutritional and environmental requirements. However, later studies reported that fastidiousness is mostly, or complementarily, related to an intrinsic slow growth (13, 14) which could not be overcome by optimized culture medium (13).

Xylella fastidiosa is the infectious agent of several diseases affecting plants of agronomical interest (15). This threatening bacterium is transmitted through xylem-sap-feeding insect vectors (15). Once inside the xylem capillary vessels, X. fastidiosa spreads, grows, produces virulence factors like exopolysaccharides (EPS), and forms biofilms (15, 16) before colonizing the foregut of a new insect host (15). X. fastidiosa’s fastidious growth was reported both *in vitro* and *in planta* and is responsible for its difficult isolation (17). Design of synthetic growth medium was carried out (18–20), but after nearly 2 decades of medium optimization, the persisting difficulty of growing X. fastidiosa, with a doubling time in the range of 100 h for some strains (19, 21), brings out that the slow growth is a property of the organism. As the genome size of the bacterium is severely reduced, it is tempting to speculate that part of the metabolic robustness is lost in X. fastidiosa. To understand the metabolic factors at the origin of fastidious growth, and the level of robustness of the bacterium, we reconstructed a high-quality genome-scale metabolic network of X. fastidiosa, based on genomic and experimental data. This network allowed us to explore the metabolic capabilities of X. fastidiosa. We unraveled a lack of metabolic robustness to sustain growth through flux variability, enzyme redundancy, gene deletion, and trophic capabilities, supported by both modeling and experiments. We then sought to deduce some metabolic determinants of fastidious growth. We found that loss of robustness mostly affected efficient parts of the network, such as enzymes with high catalytic activities, or central reactions which are assumed to play a crucial role in growth. Thereby, we unraveled that X. fastidiosa metabolism mostly relies on pathways and reactions which cannot ensure a fast proliferation. We demonstrated that the global network has a weak metabolic yield in producing central virulence factor, thus showing that it is structurally inefficient, which could also severely impair growth.

## RESULTS

### The metabolic network of X. fastidiosa.

We built a genome-scale metabolic network of X. fastidiosa (see Data Set S1, Text S1, and Text S2 in the supplemental material) using in-house automatic reconstruction algorithms (22) from the genomic sequence of Xylella fastidiosa subsp. *multiplex* strain CFBP 8418 isolated in 2015 in Corsica (23). A manual curation of each reaction was then performed using databases, literature, and simulations. Novel reactions were written to take into account the biosynthesis of EPS and lipopolysaccharides (LPS) (24–26). To represent the global cost of excreted proteins, which are crucial virulence factors, we included reactions for the biosynthesis and excretion of a proteic virulence factor through the type II secretion system (27). The reconstruction process is summarized in Data Set S2.

10.1128/mSystems.00698-19.1 TEXT S1 Metabolic network of X. fastidiosa CFBP 8418. Download Text S1, TXT file, 2.5 MB. Copyright © 2020 Gerlin et al. 2020 Gerlin et al. This content is distributed under the terms of the Creative Commons Attribution 4.0 International license.

10.1128/mSystems.00698-19.2 TEXT S2 Protocol used for generation and manual curation of the network. Download Text S2, PDF file, 0.4 MB. Copyright © 2020 Gerlin et al. 2020 Gerlin et al. This content is distributed under the terms of the Creative Commons Attribution 4.0 International license.

**FIG 1:**
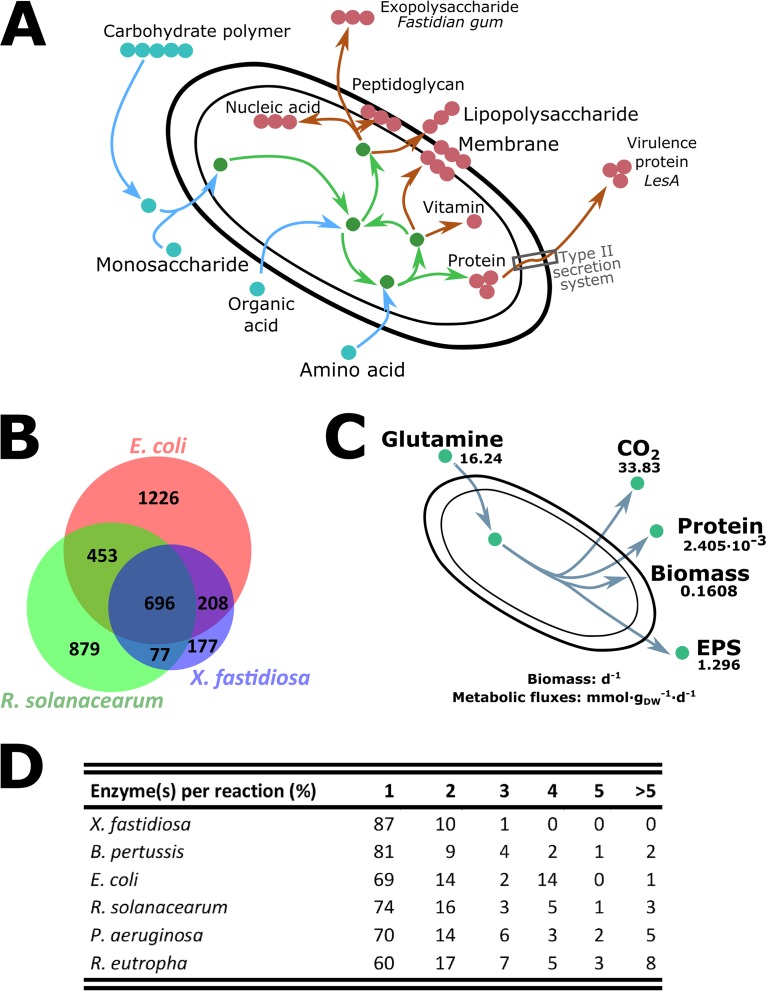
General characteristics of the reconstructed metabolic network. (A) Global overview of the reconstructed metabolic pathways in Xylella fastidiosa. This schema highlights the metabolic behaviors of Xylella fastidiosa. For catabolism (external substrates and catabolic reactions in blue), X. fastidiosa is able to degrade polymers and assimilate them or catabolize elements from xylem sap such as amino acids and organic acids. Central metabolites and reactions are schematized in green (glycolysis and TCA cycle). Metabolites/reactions in red/brown highlight anabolic capabilities: the production of biomass macromolecules (membranes, lipopolysaccharides, peptidoglycans, vitamins, proteins, and nucleic acids) and the secretion of virulence factors (virulence protein through the type II secretion system and fastidian gum [EPS]). (B) Venn diagram depicting the number of metabolic reactions in E. coli, R. solanacearum, and X. fastidiosa. The lists of the reactions found in each part of the Venn diagram are available in Data Set S2. (C) Inputs and outputs of an FBA simulation of X. fastidiosa growth on glutamine under conditions close to experimental conditions. The objective function of FBA was minimization of glutamine import. Growth and production rates were calculated from previous publications (19, 82). Flux values are in mmol·g (DW)^−1^·day^−1^ for all inputs/outputs except for biomass (day^−1^). See Text S3 for the constraints used for the simulation and Data Set S2 for the FBA solution. Scripts are available on https://github.com/lgerlin/xfas-metabolic-model. (D) Distribution of the number of enzymes for each metabolic reaction in X. fastidiosa and metabolic models from other bacteria. Proportions of reactions carried out by 1, 2, 3, 4, 5, and >5 enzymes were computed for each metabolic model. The complete data are available in Data Set S2.

The final numbers of reactions and metabolites of the X. fastidiosa network (Table 1) are remarkably small compared to Escherichia coli and R. solanacearum. However, all the core carbon and nitrogen reactions from the central metabolism were found (except one enzyme; see below), as well as the biosynthesis of all vital compounds (Fig. 1A). The precursors for the specific macromolecules EPS and LPS, which are key virulence determinants (28), can also be synthesized.

**TABLE 1:** Comparison of metabolic networks

Network characteristic	No. of reactions	No. of metabolites
E. coli	2,583	1,805
R. solanacearum	2,644	2,574
B. pertussis	1,203	1,143
X. fastidiosa	1,158	1,107

10.1128/mSystems.00698-19.3 TEXT S3 Constraints used on metabolic modeling of Xylella fastidiosa. Download Text S3, PDF file, 0.3 MB. Copyright © 2020 Gerlin et al. 2020 Gerlin et al. This content is distributed under the terms of the Creative Commons Attribution 4.0 International license.

We performed genome sequence comparisons between X. fastidiosa subsp. *multiplex* strain CFBP 8418, Xylella fastidiosa subsp. *pauca* strain 9a5c (isolated from citrus in Brazil) (29), and Xylella fastidiosa subsp. *fastidiosa* strain Temecula1 (isolated from grapevine in California) (30) to estimate the metabolic network conservation between the three subspecies (Table S1 and Table S2). Because these differential reactions represented 1% or less of the network, with most of them being not connected to central metabolism, we concluded that the architecture of the metabolic networks is probably highly similar between these three X. fastidiosa subspecies. Therefore, the conclusions drawn in the following sections on strain CFBP 8418 extend to at least three subspecies of X. fastidiosa.

10.1128/mSystems.00698-19.5 TABLE S1 Comparison of the reconstructed network of X. fastidiosa CFBP 8418 with a draft network of X. fastidiosa 9a5c. Download Table S1, PDF file, 0.6 MB. Copyright © 2020 Gerlin et al. 2020 Gerlin et al. This content is distributed under the terms of the Creative Commons Attribution 4.0 International license.

10.1128/mSystems.00698-19.6 TABLE S2 Comparison of the reconstructed network of X. fastidiosa CFBP 8418 with a draft network of X. fastidiosa Temecula1. Download Table S2, PDF file, 0.6 MB. Copyright © 2020 Gerlin et al. 2020 Gerlin et al. This content is distributed under the terms of the Creative Commons Attribution 4.0 International license.

### Predicted metabolic capacities of X. fastidiosa.

The comparison of the number of metabolic reactions shared between X. fastidiosa, E. coli, and R. solanacearum revealed that X. fastidiosa has undergone a strong metabolic reduction, which is associated with its reduced genome size, and illustrated by the decreased numbers of reactions that are specific to X. fastidiosa (Fig. 1B). The complete list of shared or specific reactions is available in Data Set S2. The metabolic core shared between the three organisms was around 700 reactions, which represents 58% of the X. fastidiosa model. E. coli shares a wide range of reactions with X. fastidiosa, including vitamin biosynthesis and transport processes. X. fastidiosa and R. solanacearum have fewer reactions in common, probably due to their more distant phylogenetic relationship (beta- versus gammaproteobacteria). Among them, we identified several metabolic pathways related to *in planta* lifestyle and pathogenicity such as nitrilase (EC 3.5.5) reactions, which detoxify nitriles (31), and peroxidases (EC 1.11.1) degrading radical oxygen species (ROS) (32), both nitriles and ROS being produced by plants upon infection (32, 33). These two plant pathogens also shared degradation reactions for plant polymers like pectin.

The comparative analysis revealed a large number of reactions (450) absent only in X. fastidiosa. Several of them are related to lipid metabolism, such as the pathway to use lipid as carbon source (β-oxidation) and to alternate carbon metabolism reactions, which enable the use of diverse compounds as carbon source, like carbohydrates (e.g., tagatose and glycogen) or small organic molecules (e.g., benzoate, d-galacturonate, and phenylacetate). These findings highlighted that X. fastidiosa metabolism appears limited in terms of metabolic diversity.

Although X. fastidiosa possesses all the vital vitamin and cofactor biosynthetic pathways, it lacks several related pathways that are generally found in fast-growing bacteria. For instance, the couple ubiquinone-8/ubiquinol-8 was the only redox couple from the quinone group found to be synthesized. In contrast to R. solanacearum, no cobalamin (vitamin B_12_) biosynthesis is possible. Accordingly, X. fastidiosa uses only enzymes with no requirement for cobalamin, which cannot be found in plants (34). We observed a similar case for molybdopterin, which forms the molybdenum cofactor.

Previous studies suggested that X. fastidiosa was limited in aerobic respiration, because no cytochromes with a high affinity for O_2_ were found in the genome sequences (35). The absence of cytochrome *c* oxidase was also reported, limiting the aerobic respiration to cytochrome *bo*_3_ ubiquinone oxidase (EC 1.10.3.10). It was thus suggested that the bacterium preferentially uses anaerobic respiration (35). Contrary to this hypothesis, no complete anaerobic respiration was found to be functional in the metabolic network, which supports the view of a functional and favored aerobic respiration in X. fastidiosa. However, the limitation of aerobic respiration suggests that the bacterium is also limited in its respiratory system.

Succinate dehydrogenase (EC 1.3.99.1), an enzyme from central and respiratory metabolism composed of four subunits, was also found to have lost a membrane subunit in X. fastidiosa. This enzymatic complex was reported to be functional with only one membrane domain (36). We thus expect that the metabolic reactions can occur in X. fastidiosa but might also be less efficient than the complete form of the enzyme complex.

Our primary analysis of the network structure showed that the core metabolism is complete, and its functionality was confirmed by flux balance analysis (FBA) simulations (Fig. 1C and Data Set S2), accepting the fact that an FBPase activity exists (see below). Simulation (Data Set S2) results show that all biomass components can be produced from the tested carbon source, glutamine, a major component of xylem sap (37–40). The majority of glutamine uptake is converted into glutamate (95% of carbon uptake) and then into α-ketoglutarate. α-Ketoglutarate is then converted through the citric acid cycle (tricarboxylic acid [TCA] cycle) to malate and oxaloacetate. Yet, the TCA cycle is not cyclic. The branch converting oxaloacetate to citric acid is not used, which could be expected for growth in minimal medium with glutamine as sole carbon source. The part of the citric acid cycle used generates central metabolites such as succinyl coenzyme A (CoA) and oxaloacetate. Pyruvate is generated from the citric acid cycle metabolite oxaloacetate, through oxaloacetate decarboxylase (EC 4.1.1.3, 22% of carbon uptake) and malic enzyme (EC 1.1.1.40, 34% of carbon uptake). Finally, these central metabolites are used to generate biomass and virulence factors (EPS and virulence proteins), and the remaining carbon is wasted during metabolic processes as CO_2_, more than half through the TCA cycle. NADH is also mainly synthesized thanks to the TCA cycle and ATP through oxidative phosphorylation.

The core central metabolism from X. fastidiosa appears to be conserved and functional, although it seems to have lost several pathways compared to other bacteria. These losses do not affect the survival of the bacterium but tend to restrict it to a limited diversity of metabolic behaviors, and probably to a slow and inefficient growth.

### Unexpected features in X. fastidiosa metabolism.

A deeper analysis of the X. fastidiosa metabolism revealed the surprising lack of some enzymes which are generally ubiquitous in bacteria. First, no gene was identified to code for fructose-1,6-bisphosphatase (FBPase) (EC 3.1.3.11), despite intensive homology searches with query sequences from a wide range of organisms. The absence of the corresponding gene in strain CFBP 8418, as well as all the other available genomes of X. fastidiosa, suggested that these bacteria are not able to perform gluconeogenesis. We verified *in silico* that in our metabolic network, gluconeogenesis was not functional; a precursor such as glutamine could not be converted into glucose with no FBPase. It would imply that X. fastidiosa can grow only in the presence of a hexose or a hexose polymer (41, 42), but that was invalidated experimentally (see below and Table 2). Therefore, conversion of fructose-1,6-bisphosphate into fructose-6-phosphate must be achieved by an alternative and previously uncharacterized route.

**TABLE 2:** *In silico* and *in vivo* growth assays on different carbon sources using FBA and Biolog PMs

Substrate	Growth assessment		Presence of compound in xylem^*c*^	
	*In silico*^*a*^	Exptl^*b*^	Grapevine	Olive tree
Amino acids				
l-Proline	+	++	Minor	Minor
l-Glutamate	+	+	ND	Minor
l-Aspartate	+	+	ND	Minor
l-Alanine	+	+	Minor	Minor
l-Glutamine	+	+	Major	Major
l-Arginine	+	+	Minor	Minor
l-Histidine	+	+	Minor	ND
l-Ornithine	+	+	ND	ND
γ-Aminobutyric acid	+	+	ND	Minor

Organic acids				
Acetic acid	+	+	ND	Minor
Citric acid	+	+	Major	ND
Pyruvic acid	+	++	ND	ND

Monosaccharides				
d-Glucose	++	++	Minor	Major
d-Galactose	++	++	ND	ND
d-Trehalose	++	++	ND	ND
d-Fructose	++	+	Minor	Major
d-Ribose	+	+	ND	ND
d-Mannose	++	+	ND	ND
d-Xylose	+	+	ND	ND

Others				
Chitin	++	++/+*	ND	ND
Dextrin	++	+	ND	ND
*myo-*Inositol	+	++	ND	Minor
Glycerol	++	++	ND	ND
d-Glucosamine	++	+	ND	ND
3-*O*-β-d-Galactopyranosyl-d-arabinose	Unknown	+	ND	ND
dl-α-Glycerol phosphate	Unknown	+	ND	ND

a Relative *in silico* growth rates were determined by computing the ratio between the growth rate and the highest growth rate obtained (on glycerol). If the relative *in silico* growth rate was >75%, growth was assessed as relatively fast (++), and if the relative *in silico* growth rate was ≤75%, growth was assessed as relatively slow (+).

b Growth assessment was made using Biolog PM respiration rate: ++ for relatively fast growth, referring to a respiration rate above 0.125 h^−1^, and + for relatively slow growth, referring to a respiration rate under 0.125 h^−1^ (see Materials and Methods and Data Set S3), except for chitin (*), for which growth was previously determined experimentally (45). Growth was observed with Biolog PMs on d,l-α-glycerol phosphate and 3-*O*-β-d-galactopyranosyl-d-arabinose but not modeled, because there are no assimilation reactions for these substrates in available metabolic models. Substrates with indeterminate growth or no growth are listed in Data Set S3.

c Xylem fluid composition from grapevine and olive tree was extracted from the work of Andersen and Brodbeck (37) and Montes Borrego et al. (39). Major, concentration ≥ 500 μM; minor, concentration < 500 μM; ND, not detected.

Strikingly, the pentose phosphate pathway also lacks two of its enzymes: the 6-phosphogluconate dehydrogenase (EC 1.1.1.44) and the transaldolase (EC 2.2.1.2). The absence of 6-phosphogluconate dehydrogenase was already reported in other prokaryotes (43), and its product ribulose 5-phosphate could be rescued through the Entner-Doudoroff pathway. However, the absence of the transaldolase is more surprising, since the enzyme is well conserved in prokaryotes (43).

### Experimental validation of carbon source usage.

In order to validate the metabolic model predictions, we experimentally assessed the growth of X. fastidiosa on 190 potential carbon (C) sources using Biolog phenotype microarrays (PMs); 8 can provide relatively fast growth and 18 can provide relatively slow growth at the scale of X. fastidiosa fastidious growth (Table 2). Growth is achievable on diverse xylem fluid components, including five amino acids, citric acid, and d-fructose, even though, surprisingly, eight compounds leading to fast growth are not major components. The number of carbon sources identified using Biolog PMs was 25, which is considerably lower than the number of C sources identified in R. solanacearum and E. coli (respectively, 36 and 86) (22, 44), confirming our prediction of reduced metabolic diversity. We also used FBA to predict the relative growth rate of X. fastidiosa on several substrates. Relative *in silico* growth rates were determined by computing the ratio between the growth rate and the highest growth rate obtained (on glycerol). The discrepancies observed between experimental and simulation results (growth *in vivo* but not predicted *in silico*) were used to correct the network, by adding the missing metabolic reactions for 12 substrates (as specified in the final metabolic network in Data Set S1). The refined metabolic network predicted growth on 24 of the 26 experimentally verified substrates; growth was observed with Biolog PMs on d,l-α-glycerol phosphate and 3-*O*-β-d-galactopyranosyl-d-arabinose but not modeled because there are no assimilation reactions for these substrates in available metabolic models. Based on the FBA results, we classified the different carbon sources (Table 2 and Data Set S3) by defining relatively fast growth *in silico* as >75% of the maximal growth rate. Sixteen of 23 (70%) of the predictions were in accordance with the experimental measurements. The highest predicted growth rate was observed with glycerol, and this was confirmed experimentally.

10.1128/mSystems.00698-19.7 DATA SET S1 Metabolic network of X. fastidiosa CFBP 8418. Download Data Set S1, XLSX file, 0.2 MB. Copyright © 2020 Gerlin et al. 2020 Gerlin et al. This content is distributed under the terms of the Creative Commons Attribution 4.0 International license.

10.1128/mSystems.00698-19.8 DATA SET S2 Network analysis and modeling. General network composition, biomass objective function, comparative analysis, FBA results, genes and enzymes per reaction distribution, FVA results, efficiency analysis, study of FBPase inefficiency. Download Data Set S2, XLSX file, 0.2 MB. Copyright © 2020 Gerlin et al. 2020 Gerlin et al. This content is distributed under the terms of the Creative Commons Attribution 4.0 International license.

10.1128/mSystems.00698-19.9 DATA SET S3 Carbon source study: Biolog PM results, *in silico* growth assays on different carbon sources, and xylem fluid chemistry. Download Data Set S3, XLSX file, 0.9 MB. Copyright © 2020 Gerlin et al. 2020 Gerlin et al. This content is distributed under the terms of the Creative Commons Attribution 4.0 International license.

We also tested *in silico* the growth on chitin, a polymer composing the insect foregut wall, not present on Biolog PM plates. A recent study showed the necessity of a chitinase (ChiA) for both insect and plant colonization and suggested that chitin is the favored carbon source in the insect environment (45). Our results (Table 2) support the hypothesis that chitin is a favored substrate since it provides some of the fastest growth (relatively speaking for X. fastidiosa).

### Modeling-based evidence for a fragile metabolism.

To first estimate the level of functional redundancy in the X. fastidiosa network, we determined the number of enzymes associated with each reaction in comparison with other reference metabolic models (Fig. 1D and Data Set S2). Eighty-seven percent of the metabolic reactions in X. fastidiosa are carried out by a unique enzyme, while no other organism exceeds 80% except the also-fastidious Bordetella pertussis (11). Alternative enzymes are indicative of functional redundancies in the network and are generally associated with environmental plasticity. This lack in X. fastidiosa illustrated further the low flexibility of its metabolism. We then performed a gene essentiality analysis on the network. The results for X. fastidiosa and other reference organisms are presented in Table 3 (detailed information in Data Set S4). Strikingly, the predicted proportion of essential genes was 54% on glucose and 51% in a protein-rich environment, which is considerably higher than the other tested bacteria. This outstandingly high proportion of essential genes in X. fastidiosa illustrates the fragility of the metabolic network against perturbations.

**TABLE 3:** Simulation analyses of the metabolic network

Organism	Proportion (%) of:			
	Essential metabolic genes (gene essentiality^*a*^) in:		Reactions (flux variability^*b*^)	
	Glucose medium	Protein-rich environment	Varying	Bidirectional
E. coli	15	12	60	4
R. solanacearum	15	13	48	3
B. pertussis	No growth	40		
X. fastidiosa	54	51	32	1

a Proportion of essential metabolic genes according to metabolic modeling, in glucose medium and a protein-rich environment. As B. pertussis cannot grow on glucose, the gene essentiality value is given only for a simulated protein-rich growth environment (12). Data for B. pertussis and E. coli (growth on glucose) were extracted from the work of Fyson et al. (12) and Orth et al. (67). Values for R. solanacearum and X. fastidiosa in both environments and for E. coli in a protein-rich environment were computed for this study. The detailed results are available in Data Set S4.

b Assessment of flux variability. The proportion of varying reactions represents the number of reactions with non-null flux variation divided by the total number of metabolic reactions in the network. Bidirectional reactions are reversible reactions that can carry out both positive and negative flux to sustain an optimal growth. The proportion was equally determined by calculating the ratio of these reactions divided by the total number of metabolic reactions in the network. The detailed results are available in Data Set S2.

10.1128/mSystems.00698-19.10 DATA SET S4 Gene and reaction essentiality. Download Data Set S4, XLSX file, 0.3 MB. Copyright © 2020 Gerlin et al. 2020 Gerlin et al. This content is distributed under the terms of the Creative Commons Attribution 4.0 International license.

Fragility and low flexibility of X. fastidiosa metabolism were confirmed using flux variability analysis (FVA). FVA is an *in silico* analysis to estimate if alternative flux values, due to an internal or external perturbation, will still sustain an optimal metabolic behavior. We performed FVA using l-glutamine as carbon source and growth as the objective function. Similar analyses were also conducted with E. coli and R. solanacearum (Table 3 and Data Set S2). As expected from the previous results, the proportion of varying reactions (i.e., reactions with alternative fluxes able to sustain the biological objective) was significantly lower (32%) in X. fastidiosa than in R. solanacearum (48%) and E. coli (60%). Thus, only a limited proportion of the metabolic reactions can still sustain optimal growth if their fluxes vary. This particular trait confirmed a lack of flexibility in X. fastidiosa metabolism.

### A higher cost for virulence can compromise X. fastidiosa growth.

We finally assessed the efficiency of the network structure for a specific task by determining metabolic yields on proliferation and on virulence factors. Metabolic yields were defined as the maximal proportion of carbon which could be invested in the biosynthesis of a specific macromolecule (virulence factor) or biomass. The obtained efficiency data are presented in Table 4 and compared to another plant pathogen, R. solanacearum. This other plant pathogen also has a network including virulence proteins and EPS secretion in the model, in contrast to other metabolic models including E. coli. The comparison was thus limited to these two plant pathogens.

**TABLE 4:** Structural efficiency analysis of X. fastidiosa and R. solanacearum metabolic models^*a*^

Biological objective	Yield (%) for species:	
	Xylella fastidiosa	Ralstonia solanacearum
Biomass	54	58
Protein	61	64
EPS	58	72

a Metabolic yields were compared for different biological objectives: (i) production of biomass, (ii) production of virulence proteins, and (iii) production of EPS. The mathematical details on yield determination are given in Data Set S2.

Similar efficiency is observed for biomass production and extracellular protein secretion (Table 4; Data Set S2). However, the structure of the X. fastidiosa metabolic network is less efficient for production of EPS than R. solanacearum (Table 4 and Data Set S2), with an increase of losses as CO_2_ (+16%). In other words, biosynthesis of this virulence factor has a higher carbon cost in X. fastidiosa and this probably impairs reallocation of carbon fluxes to sustain efficient biomass production.

## DISCUSSION

Fastidious growth is a paradoxical property of some prokaryote pathogens, and we sought to understand the emergence of this phenotype through a metabolic network study in a particularly fastidious organism, the plant pathogen X. fastidiosa. This bacterium appears to possess a minimal but fully functional metabolic network, and this was confirmed experimentally. This is in contrast with several other pathogen or symbiont species which have also undergone reductive evolution with the loss of essential metabolic functions (12, 46, 47).

### The reduction of the X. fastidiosa metabolic network probably results from its adaptation to a limited number of environments.

The reduction of the metabolic network does not create any auxotrophy for X. fastidiosa growth but tends to restrict the pathogen to a limited diversity of metabolic behaviors. X. fastidiosa is specifically restricted to two environments (plant vascular vessels and the foregut of xylem-eating insects) (15). These environments have common features as they are both constituted of xylem sap and carbonated polymers (plant cell wall and chitin), and it has been shown that the chitin-degrading enzyme is essential for colonization both in the insect and *in planta* (45). Thereby, X. fastidiosa appears to be restricted to a homeostatic environment, i.e., overall constant in composition and not prone to external perturbations. This lifestyle restricted to specific environments probably explains the limitation of metabolic behaviors (particularly the catabolic capacities), which was inferred from our study.

One could argue that fastidiousness of *Xylella* may be due to an environment poor in nutrients. However, *Xylella* has a fastidious growth even in nutrient-rich artificial media (e.g., buffered charcoal yeast extract [BCYE] medium [21]). In addition, other vascular plant pathogens such as R. solanacearum have a high proliferation rate in the same environment: 3 days after injection in stems, R. solanacearum can reach up to 10^9^ CFU per gram of fresh weight (48). Thereby, slow growth cannot be explained by a nutritional limitation *in planta* but seems to be due to intrinsic specificities of the pathogen.

### The genome-scale metabolic analysis reveals a lack of metabolic robustness.

Robustness is a key feature of biological systems, which allows them to maintain their function(s) when subjected to environmental or internal perturbation (7, 49). Genome-scale analysis of X. fastidiosa metabolism was carried out to examine if robustness could have shaped the evolution of its ancestor. By studying gene essentiality and enzyme distribution, we showed that functional redundancy, one of the main sources of robustness, is substantially missing in the X. fastidiosa metabolic network. This lack of robustness reduces the possibilities for X. fastidiosa to protect itself against perturbations both internal as mutations and external as environmental perturbations. FVA showed that the network lacks flexibility, and as mentioned above, this lack of robustness is also characterized by restricted trophic capabilities. This global fragility certainly makes X. fastidiosa very sensitive to deleterious mutations. The high level of homologous recombination observed in the species (50, 51) could serve as a rescue mechanism in this context.

### Metabolic peculiarities responsible for a slow-growth phenotype.

**FIG 2:**
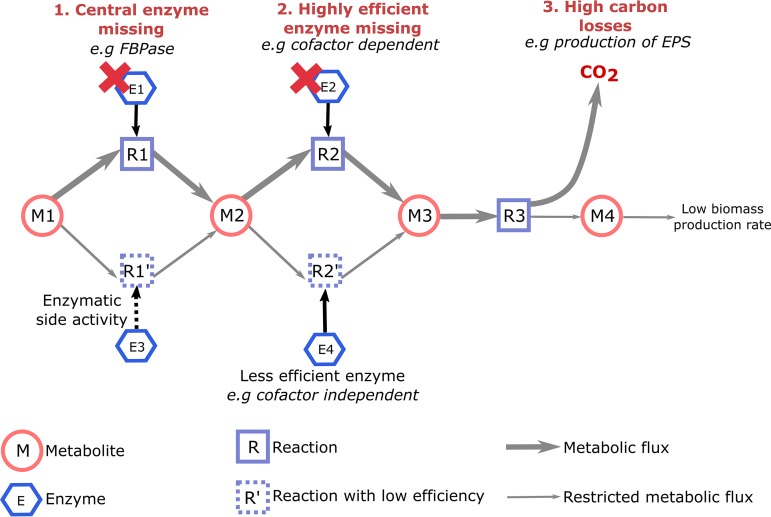
Metabolic properties contributing to fastidious growth. The three main metabolic properties unraveled by our study and probably contributing to fastidious growth are listed in red at the top of the figure. These three properties limit the metabolic fluxes. For 1 and 2, as the highly efficient enzyme is not available, a less efficient enzyme performs the reaction, which decreases the metabolic fluxes. For 3, the production of virulence factor strongly enhances carbon losses as CO_2_, which will limit the ability of the network to efficiently convert substrate into biomass. These flux limitations are predicted to strongly reduce growth rate and be responsible, possibly with additional inputs such as regulation, for fastidious growth.

Several elements were raised by our study (Fig. 2).

### (i) X. fastidiosa metabolism lacks certain genes/pathways which are not responsible for auxotrophy but are nevertheless assumed to ensure rapid growth.

Some peripheral biosynthetic pathways appear to be missing, such as the ones for cobalamin and molybdopterin. It limits the bacterium to cobalamin- and molybdopterin-independent enzymes, usually possessing a lower enzymatic activity (52, 53). Similar observations were made on the respiratory metabolism, which appears to be restricted to a combination of poorly efficient cytochrome and cytochrome oxidase. A central enzyme, succinate dehydrogenase, also involved in respiratory metabolism, surprisingly lacks a membrane subunit, which could also affect the efficiency of central metabolic fluxes.

Furthermore, X. fastidiosa lacks two key enzymes: fructose-1,6-bisphosphatase (FBPase) for gluconeogenesis and transaldolase in the pentose phosphate pathway. We found that these lacks were not deleterious, suggesting that for FBPase the reaction was processed by a nonidentified route. The emerging concept of underground metabolism provides a reasonable hypothesis: the missing reaction could be achieved through the side activity of another enzyme, which leads to a reduced enzymatic activity (54).

A reduction in enzymatic activity in a reaction central for growth is assumed to strongly reduce the proliferation rate. To illustrate this phenomenon, we estimated that the experimentally observed X. fastidiosa growth rate (0.1608 day^−1^) (19) was recovered *in silico* with a 3.37-fold reduction of the optimal FBPase flux (see Data Set S2 in the supplemental material). This reduction converted the generation time from 1.45 h (typical optimal bacterial growth) to 103 h (fastidious growth), so the growth rate is reduced by 71-fold, while the FBPase flux is reduced by only 3.37-fold. This *in silico* reduction seems reasonable, as higher flux reductions were experimentally observed (see, for example, a 200-fold reduction for an enzymatic activity in reference 54).

In photosynthetic organisms, a double role of sedoheptulose-1,7-bisphosphatase (SBPase) and FBPase activity in the same enzyme was reported, reinforcing the view that FBPase activity could be achieved by an alternative enzyme, with a lower efficiency (55–57). No SBPase exists in nonphotosynthetic organisms, but we hypothesize that another enzyme, *myo-*inositol 1-phosphatase (IMPase), could have a bisphosphatase activity on fructose-1,6-bisphosphate. This IMPase enzyme (EC 3.1.3.25) was identified in the X. fastidiosa metabolic network. Although *myo-*inositol is not a hexose, it is a 6-carbon cyclic molecule with hydroxyl groups, which makes its chemical structure very similar to fructose. An enzyme with dual IMPase/FBPase activity was found in archaea (58) and also in the slow-growing pathogen Mycobacterium tuberculosis (59). It was shown that the archaeal IMPase had a catalytic activity on fructose-1,6-bisphosphate 3-fold lower than a classical FBPase (21 s^−1^ versus 7 s^−1^) (58). Remarkably, the difference of activity measured in that study (3-fold) is similar to the loss of enzymatic efficiency we predicted to match fastidious growth (reduction of flux: 3.37-fold).

The loss of FBPase is observed among all X. fastidiosa strains sequenced to date. This observation suggests that this enzyme was already lost in the *Xylella* ancestor, whereas this enzyme (EC 3.1.3.11) is broadly conserved in living organisms (60). Looking more closely at the *Xanthomonadaceae* phylum to which *Xylella* belongs, it appears that FBPase is conserved in all *Xanthomonas* species except Xanthomonas albilineans. Interestingly, *X. albilineans* is also a xylem-limited and slow-growing plant pathogen that has undergone reductive evolution, although less extensively than X. fastidiosa (61). Future investigations should address whether the loss of FBPase is linked to a specialization in terms of environment or lifestyle.

### (ii) X. fastidiosa metabolism is structurally inefficient.

The decreased metabolic efficiency (in terms of yield) was particularly obvious in comparison with another vascular plant pathogen, R. solanacearum, when we determined the cost for the production and excretion of EPS, a major virulence factor of these bacteria (24, 62). In R. solanacearum, it was shown that EPS production has a significant metabolic cost that can impact biomass production (loss of approximately 25% of the growth rate due to EPS production) (22). Our simulations (Table 4) predict that the cost for EPS production is an even higher burden for X. fastidiosa.

Fastidious growth probably results from a combination of two factors. First is the lack of reactions catalyzed by efficient enzymes, cobalamin/molybdopterin-dependent enzymes, complete succinate dehydrogenase complex, efficient respiratory chain, and, strikingly, the absence of a central enzyme in gluconeogenesis. Second is a global inefficiency in producing virulence factors. We cannot formally exclude that enzymatic efficiency could be globally lower in *Xylella* species than in other bacteria. Surprisingly, the predictions from the efficiency analysis indicate that the potential for biomass production by X. fastidiosa is virtually similar to that of R. solanacearum (Table 4). This observation suggests that constraints imposed on metabolic efficiency both by virulence factor production and by regulation patterns specifically limit the growth of X. fastidiosa. Supporting this hypothesis, it was reported that an X. fastidiosa mutant deficient for the production of a secreted and virulence-related protein had a growth rate significantly increased in comparison to the wild-type strain (63). In such a scenario, the fastidious behavior could be viewed as a developmental strategy of the pathogen to remain at a low population level inside the host and avoid detection by its immune system. This hypothesis is in agreement with the view of *Xylella* as a “self-limiting” organism, which has many features of a plant commensal but accidentally provokes epidemics due to an exaggerated and late plant response (64), probably linked to specific environmental conditions.

### Common metabolic peculiarities in fastidious pathogens.

It is interesting to put these findings in relation to other fastidious organisms. The pathogen B. pertussis, similarly to X. fastidiosa, experienced a strong genome reduction, which implied a metabolic network reduction, leading to a number of reactions and metabolites close to X. fastidiosa (Table 1). The fastidious growth of B. pertussis could thus also be related to a lack of functional redundancy favoring less efficient pathways, and it is also conceivable that this reduction could affect the network efficiency and increase the cost of virulence factor production. Furthermore, another core metabolic enzyme is missing in B. pertussis, making its glycolysis unachievable (12). In contrast to X. fastidiosa, this absence led to an auxotrophy for amino acids, but one can consider that this absence also constrains the metabolic fluxes and provokes fastidious growth. Another fastidious pathogen, M. tuberculosis, has an interesting similarity to X. fastidiosa; the lack of standard fructose-1,6-bisphosphatase was also reported in the pathogen, suggesting the use of an enzyme side activity (59) or an atypical fructose-1,6-bisphosphatase enzyme (65). These common metabolic particularities between slow-growing pathogens might indicate that in an environment where fast growth could activate the host immune system, similar evolutionary processes of growth reduction, at different degrees and strengths, might favor the emergence of fastidious phenotypes.

In conclusion, based on genetic and phenotypic data and computational approaches, we were able to unravel several candidate genes responsible for bacterial fastidious growth. A perspective of this study is to validate these candidates by functional genetic approaches to establish their involvement in the fastidious growth phenotype.

## MATERIALS AND METHODS

### Bacterial strain.

Analyses were conducted with the strain CFBP 8418, an X. fastidiosa subsp. *multiplex* ST6 (sequence type 6) strain isolated from Spartium junceum in Alata (France) (23) and provided by the French Collection for Plant-associated Bacteria (CIRM-CFBP).

### Carbon substrate phenotyping.

Phenotyping was performed using Biolog phenotype microarray plates PM1 and PM2A according to the manufacturer’s protocol, except that Tween 40 was replaced by Tween 80. An initial optical density (OD) of 0.18 (600 nm) was used for inoculation. Incubation time was 72.5 h. Three independent replicates were performed.

Growth was assumed proportional to respiration and was assessed by calculating the area under the curve (AUC) and the slope of the logarithm on the exponential phase (respiration rate). Growth was considered effective if AUC was >AUC_negative control_ + threshold (see Data Set S3). If the respiration rate was above 0.125 h^−1^, the growth was categorized as fast (++); otherwise, it was categorized as slow (+). If the results were nonreproducible, the growth was assessed to be undetermined. For six substrates (out of 190), for which growth was assessed unambiguously as positive for two out of the three replicates, growth was referred to (+). The complete results are available in Data Set S3.

### Genome-scale metabolic network reconstruction.

The genome-scale metabolic network reconstruction was performed from the genomic sequences of the strain CFBP 8418 (GenBank accession no. LUYA00000000.1) (23). The protocol to generate high-quality genome-scale metabolic network proposed by Thiele and Palsson (66) was followed.

For the automatic reconstruction, the genomic sequences were compared, by priority order, to the five following metabolic models: Escherichia coli K-12 MG1655 (iJO1366 [67]), Ralstonia solanacearum GMI1000 (iRP1476 [22]), Pseudomonas aeruginosa PAO1 (iMO1086 [68]), Ralstonia eutropha H16 (RehMBEL1391 [69]), and Bacillus subtilis 168 (iYO844 [70]). X. fastidiosa sequences were compared by orthology to the sequences from each metabolic model, generating five draft models, thanks to the Autograph method (71). The SAMIR tool (22) was used to reconcile the identifiers between the models. Finally, the five metabolic models were merged into one draft model expressed with BiGG identifiers (72), following the priority order stated above and depending on the orthology quality.

The draft metabolic model was manually curated, evaluating the reactions one by one. The accuracy of each reaction was checked using KEGG (60), BiGG (72), BioCyc (73), MetaCyc (73), and bibliographical searches. Simulations (see below) were performed pathway by pathway to check if the curated pathway was functional. A manual gap-filling step was processed for nonfunctional pathways, looking for evidence of the missing reactions. Each reaction was scored to assess its reliability with a specific score that was more adapted to the network than the confidence score proposed by Thiele and Palsson (66). The meaning of this specific score and the complete manual curation protocol are available in Text S2. The two scores are given for each reaction in the metabolic network (Data Set S1).

As proposed by Thiele and Palsson (66), a biomass objective function was defined. General characterizations of the X. fastidiosa genome (29) and specificities of the studied strain (23) were both used. For missing information, data from Xanthomonas campestris pv. campestris (74) and E. coli (67) were used (Data Set S2). After curation, the tool CarveMe (75) was used to find other potential reactions. The generated reactions were manually curated and added to the network.

For metabolic network comparisons between subspecies, draft metabolic networks of Xylella fastidiosa subsp. *pauca* 9a5c (GenBank accession no. GCA_000006725.1) (29) and X. fastidiosa subsp. *fastidiosa* Temecula1 (GenBank accession no. GCA_000007245.1) (30) were generated with the same reconstruction process.

### Comparative analysis.

The reaction identifiers were given as input of the comparative analysis between E. coli, R. solanacearum, and X. fastidiosa. The metabolic model considered for E. coli was the model iJO1366 (67). For R. solanacearum, the model iRP1476 (22) was used, taking into account only the metabolic module. The Venn diagram was generated using the online tool BioVenn (76). The list of reactions in each part of the diagram is available in Data Set S2.

### Computational simulations.

To test the functionality of pathways and of the global network, simulations were performed on the model. To access intracellular fluxes and growth rate, the flux balance analysis (FBA) methodology was used (77). The constraints used to model the system are detailed in Text S3. To access the variability of each flux, flux variability analysis (FVA), a methodology based upon the same principles, was then performed (78). A deviation of 1% from the optimality was allowed. *In silico* gene essentiality analysis was performed through multiple FBAs on the network. For each FBA, a metabolic gene of the network is deleted to test if its deletion has an impact on the organism growth. The *in silico* gene essentiality analysis in a protein-rich environment was inspired by a B. pertussis publication (12): l-glutamate as the major carbon source and an availability 10 times smaller in number of carbon for each other proteinogenic amino acid. For FVA and *in silico* gene essentiality, protein and EPS constraints were removed.

The structural efficiency of the network was tested in X. fastidiosa and, as a comparison, in R. solanacearum. To avoid bias, all the constraints were removed, and a generic l-glutamine uptake rate of 1 mmol**·**h^−1^**·**g (dry weight [DW])^−1^ was used. Each of the biological processes (biomass and virulence) was studied separately, and the maximization of their synthesis was used as the objective. Each simulation result gave access to the proportion of carbon lost in the form of CO_2_, with the exported CO_2_ rate (R_EX_CO2_e). This allowed us to determine the maximal amount of carbon which could be dedicated to a virulence factor or biomass production. Metabolic yields, and consequently cost of virulence factors, were computed as the ratio between maximal production rate and the uptake rate, normalized by the number of carbons.

Text S4 provides a detailed description of mathematical modeling of metabolism used in the study.

10.1128/mSystems.00698-19.4 TEXT S4 Mathematical modeling of metabolism and FlexFlux user guide. Download Text S4, PDF file, 0.9 MB. Copyright © 2020 Gerlin et al. 2020 Gerlin et al. This content is distributed under the terms of the Creative Commons Attribution 4.0 International license.

Simulations were performed with the open source tool FlexFlux (79) and are reproducible using the guide provided in Text S4. The linear programming solver CPLEX, developed by IBM, was used to solve the system and get solutions. Scripts and command lines are available online on GitHub: https://github.com/lgerlin/xfas-metabolic-model.

### Data availability.

All data generated or analyzed during this study are included in figures or tables or in the supplemental material. In particular, raw data from Biolog phenotype microarray plates (for carbon substrate phenotyping) are available in Data Set S3.

### Model and code availability.

The X. fastidiosa subsp. *multiplex* strain CFBP 8418 metabolic model was named Xfm1158. The metabolic model was deposited in BioModels (80) and assigned the identifier MODEL2003100001. Exploration, omics mapping, and basic flux analyses can be performed on the model in MetExplore (81) (https://metexplore.toulouse.inrae.fr/metexplore2/?idBioSource=5822). The main scripts and command lines used for the study are available on GitHub: https://github.com/lgerlin/xfas-metabolic-model.
