# Supplementary material for: Genome-Scale Investigation of the Metabolic Determinants Generating Bacterial Fastidious Growth
Source: mSystems. 2020 Mar 31;5(2):e00698-19. doi: 10.1128/mSystems.00698-19 (PMC7112962; doi:10.1128/mSystems.00698-19)
Supplement: TEXT S2 [file mSystems.00698-19-s0002.pdf]

Xylella fastidiosa metabolic network curation

I. Curation process

The network was curated pathway by pathway, starting by central and canonical pathways.

For each reaction in a pathway:

- Name, ID, FormulaIds, FormulaNames, EC, pathways, type were verified using KEGG, MetaCyc and BiGG. For the reversibility, MetaCyc (particularly EcoCyc) was used as reference.
- Changes were explained in comments.
- If the prop2 score = 100, there was an orthology for all the necessary genes. The reaction was kept in the network except if it was a duplicate.
- If the prop2 score was below 100, literature and metabolic databases (KEGG, MetaCyc) were used to decide if the reaction was kept. BLASTP and Conserved Domains Databases were used to find if a *X. fastidiosa* gene could complete the enzymatic complex.
- Depending on the results of this database research, a score was attributed to the reaction to represent the probability of this reaction, and it was indicated if the reaction has to be deleted or kept.

After curation, the orthology was different than the one generated by the automatic reconstruction. So, a column “final genes” and “final orthology” were added. Pathways names were manually rewritten to standardize them in the network, in the column “pathways”. Automatically generated subsystems were kept in the column “subsystem”.

When an information was missing, the mention No_Assignment was attributed.

II. Network guide

- **#Id**

Reaction ID usually come from BiGG databases. Some ID were manually written when this was a new reaction in accordance with the nomenclature.

- **FormulaIds**

The BiGG database nomenclature was used. The reversibility of the reaction was manually curated but is sometimes uncertain and should be treated cautiously.

- **EC**

EC number were verified using KEGG, as some of the EC numbers generated automatically were out-of-date. When there was no EC for a reaction, No_Assignment was attributed.

- **type**

Indicates if the reaction is internal (inside the cell) or a transport/exchange reaction, or external (outside the cell).

- **prop**

From the automatic reconstruction, indicates the proportion (%) of genes from the reference organism that were conserved.

- **prop2**

From the automatic reconstruction, indicates the proportion (%) of essential genes from the reference organism that were conserved. If the score is 100, there are all orthology evidences necessary for the reaction.

- **ref_no**

Indicates from each organism of the automatic reconstruction the orthology was found.

ref_no	strain	model
1	*Escherichia coli* K-12	iJO1366
2	*Ralstonia solanacearum* GMI1000	iRP1476
3	*Pseudomonas aeruginosa* PAO1	iMO1086
4	*Ralstonia eutropha* H16	RehMBEL1391
5	*Bacillus subtilis* 168	iYO844

- **quality score**

A score from 1 to 7 was given to assess the probability of the reaction. A score of 1 is for the reaction with high probability, and a score of 7 is for simple assumptions that would need to be studied more precisely.

Some reactions were generated using the tool fast automated reconstruction (Nucleic Acids Research, gky537, https://doi.org/10.1093/nar/gky537). As no precise information was given for the quality of the reaction, it was ranked score 3.

- **Quality score**

Our own quality score, adapted to the network.

Situation	Score	Thiele and Palsson score associated	Validation level
Complete orthology (prop score 100)	1	2	**Validated**
Spontaneous reaction	1	2	**Validated**
Sufficient orthology (prop2 score 100)	2	2	**Validated**
Evidence of the reaction in publications	3	2, 3, 4	**Validated**
Reaction generated by fast automated reconstruction	3	2, 3, 4	**Validated**
Missing gene non-essential	4	2	**Validated**
Missing gene found in the target genome	5	2	**Validated**
prop2 score ≥ 50 and reaction connected to others	6	1	**Assumed**
reaction necessary to complete a pathway	6	1	**Assumed**
Arbitrary choice, modeling	7	1	**Assumed**
Potential reaction: no evidence and no connection to the network	7	1	**Assumed**

- **Thiele and Palsson score**

Confidence score proposed by Thiele and Palsson, Nat Protoc., 2010, related to our quality score.

To choose between 2, 3, 4:

2	Physiological data (indirect evidence from secretion product, defined medium…)
3	Genetic data (knock-out, knock-in, over-expression)
4	Biochemical data (direct evidence like protein purification, biochemical assays…)

- **reference (PMID)**

The PubMed ID (PMID) was used each time a publication was used to cure a reaction: evidence of the reaction, characterization of a macromolecule, *X. fastidiosa* specificity for example.

- **final genes**

The final genes from *X. fastidiosa* generating the reaction are given. If a gene is unknown, No_Assignment was used.

- **final orthology**

The final ortholog from a reference organism (sometimes not in the list of the five reference organisms) that was used to build the relation between gene and reaction.

- **origin**

It is precised if the reaction was generated by the automatic reconstruction, was manually added or comes from fast automated reconstruction

- **pathways**

A standardized version of the pathways was manually added.
