# Supplementary material for: Genome-Scale Investigation of the Metabolic Determinants Generating Bacterial Fastidious Growth
Source: mSystems. 2020 Mar 31;5(2):e00698-19. doi: 10.1128/mSystems.00698-19 (PMC7112962; doi:10.1128/mSystems.00698-19)
Supplement: TEXT S3 [file mSystems.00698-19-s0003.pdf]

Constraints used on metabolic modeling of *Xylella fastidiosa*

The following specific constraints were used to perform simulations.

Each carbon exchange reaction was set to zero, except the carbon source used in the simulation:

R_EX_gal_e, R_EX_asp_L_e, R_EX_pro_L_e, R_EX_tre_e, R_EX_man_e, R_EX_glyc_e, R_EX_xyl_D_e, R_EX_glu_L_e, R_EX_mal_L_e, R_EX_rib_D_e, R_EX_fru_e, R_EX_ac_e, R_EX_glc_D_e, R_EX_asn_L_e, R_EX_uri_e, R_EX_adn_e, R_EX_cit_e, R_EX_inost_e, R_EX_gly_glu_L_e, R_EX_ala_L_e, R_EX_acac_e, R_EX_lyx_L_e, R_EX_pyr_e, R_EX_dextrin_e, R_EX_4abut_e, R_EX_gam_e, R_EX_arg_L_e, R_EX_his_L_e, R_EX_orn_e, R_EX_chitin_polymer_e, R_EX_gln_L_e

As no carbon uptake is known for *X. fastidiosa*, a minimal L-glutamine import (*R_EX_gln_L_e*) was put as the objective function. Growth rate and protein excretion flux were estimated from a published growth assay on defined medium (XDM2*) (Leite, Andersen, and Ishida 2004). To simplify the system, the protein excretion flux was defined as an excretion of the protein LesA, described as a major excreted product of the bacterium (Nascimento et al. 2016). EPS excretion flux was estimated from a published quantification assay (Navarrete and De La Fuente 2014). ATP maintenance was assumed to be proportional to the growth rate, so was estimated to be 3.073 $mmol.g_{DW}^{-1}.d^{-1}$, taking as reference that *R. solanacearum* ATP maintenance is 8.39 $mmol.g_{DW}^{-1}.h^{-1}$ for a growth rate of 0.439 h^{-1}.

Flux values were converted by day unit instead of hour unit since values are small. It led to the following constraints:

**Reaction**	**Flux ($mmol.g_{DW}^{-1}.d^{-1}$)**
R_DM_BIOMASS_c	0.1608
R_DM_EPS_XF_e	1.296
R_DM_LesA_e	0.002405
R_ATPM	3.0731

References

Leite, Breno, Peter Craig Andersen, and Maria Lucia Ishida. 2004. “Colony Aggregation and Biofilm Formation in Xylem Chemistry-Based Media for Xylella Fastidiosa.” *FEMS Microbiology Letters* 230(2): 283–90.

Nascimento, Rafael et al. 2016. “The Type II Secreted Lipase/Esterase LesA Is a Key Virulence Factor Required for Xylella Fastidiosa Pathogenesis in Grapevines.” *Scientific Reports* 6(January): 1–17. http://dx.doi.org/10.1038/srep18598.

Navarrete, Fernando, and Leonardo De La Fuente. 2014. “Response of Xylella Fastidiosa to Zinc: Decreased Culturability, Increased Exopolysaccharide Production, and Formation of Resilient Biofilms under Flow Conditions.” *Applied and Environmental Microbiology* 80(3): 1097–1107.
