# Supplementary material for: Genome-Scale Investigation of the Metabolic Determinants Generating Bacterial Fastidious Growth
Source: mSystems. 2020 Mar 31;5(2):e00698-19. doi: 10.1128/mSystems.00698-19 (PMC7112962; doi:10.1128/mSystems.00698-19)
Supplement: TABLE S1 [file mSystems.00698-19-st001.pdf]

Supplementary Table 1

Comparison of the reconstructed network of *X. fastidiosa* CFBP 8418 with a draft network of *X. fastidiosa* 9a5c.

Reaction ID	Reaction name	Reaction formula	EC number	Pathway	Gene
Reactions absent in 9a5c strain but identified in CFBP 8418					
R_NNAMr	nicotinamidase	1 M_h2o_c + 1 M_ncam_c -> 1 M_nh4_c + 1 M_nac_c	3.5.1.19	NAD NADP biosynthesis	(XFCFBP8418_020780)
R_SHCHD2	sirohydrochlorin dehydrogenase (NAD)	1 M_nad_c + 1 M_dscl_c -> 1 M_scl_c + 1 M_h_c + 1 M_nadh_c	1.3.1.76	Porphyrin metabolism	(XFCFBP8418_010970)
R_SHCHF	sirohydrochlorin ferrochelatase	1 M_scl_c + 1 M_fe2_c -> 1 M_sheme_c + 3 M_h_c	4.99.1.4	Porphyrin metabolism	(XFCFBP8418_010970)
R_UPP3MT	uroporphyrinogen methyltransferase	2 M_amet_c + 1 M_uppg3_c -> 1 M_dscl_c + 2 M_ahcys_c + 1 M_h_c	2.1.1.107	Porphyrin metabolism	(XFCFBP8418_010970)
R_MTAP	5'-methylthioadenosine:orthophosphate methylthio-D-ribosyltransferase	1 M_5mta_c + 1 M_pi_c -> 1 M_5mdr1p_c + 1 M_ade_c	2.4.2.28	Met Cys Ser metabolism	(XFCFBP8418_021330)
Reactions absent in CFBP 8418 strain but identified in 9a5c					
R_ALHD4	Aldehyde dehydrogenase (butanal, NAD)	1 M_nad_c + 1 M_h2o_c + 1 M_btal_c -> 1 M_nadh_c + 1 M_1boh_c + 2 M_h_c	1.2.1.57	Alternate carbon metabolism	(WP_010894208.1) or (WP_010894291.1) or (WP_038200764.1)
R_ACTNabc	R-acetoin transport via ABC system	1 M_atp_c + 1 M_h2o_c + 1 M_actn_R_e -> 1 M_adp_c + 1 M_h_c + 1 M_pi_c + 1 M_actn_R_c	NA	Alternate carbon metabolism	(WP_023906641.1)
R_ACTNabc1	R-acetoin efflux via ABC system	1 M_atp_c + 1 M_h2o_c + 1 M_actn_R_c -> 1 M_adp_c + 1 M_h_c + 1 M_pi_c + 1 M_actn_R_e	NA	Alternate carbon metabolism	(WP_023906641.1)
R_CINNDO	Cinnamate dioxygenase	1 M_cinnm_c + 1 M_h_c + 1 M_nadh_c + 1 M_o2_c -> 1 M_cenchddd_c + 1 M_nad_c	NA	Alternate carbon metabolism	(WP_010893973.1)
R_PPPNDO	Phenylpropanoate dioxygenase	1 M_h_c + 1 M_nadh_c + 1 M_o2_c + 1 M_pppn_c -> 1 M_cechddd_c + 1 M_nad_c	NA	Alternate carbon metabolism	(WP_010893973.1)
R_POAACR	Peroxyaminoacrylate reductase	1 M_nadh_c + 1 M_poaac_c -> 1 M_3amac_c + 1 M_h2o_c + 1 M_nad_c	NA	Nucleotide Salvage Pathway	(WP_010892886.1)
R_EXOGLAS	Exoglucanase	1 M_14bglucan_e + 5 M_h2o_e -> 6 M_cellb_e	3.2.1.91	Plant cell wall degradation	(WP_010893773.1)
R_GLCDpp	glucose dehydrogenase (ubiquinone 8 as acceptor)	1 M_glc_D_p + 1 M_h2o_p + 1 M_q8_c -> 1 M_h_p + 1 M_glcn_p + 1 M_q8h2_c	1.1.5.2	Oxidative phosphorylation	(WP_010894211.1)
