# Supplementary material for: Genome-Scale Investigation of the Metabolic Determinants Generating Bacterial Fastidious Growth
Source: mSystems. 2020 Mar 31;5(2):e00698-19. doi: 10.1128/mSystems.00698-19 (PMC7112962; doi:10.1128/mSystems.00698-19)
Supplement: TABLE S2 [file mSystems.00698-19-st002.pdf]

Supplementary Table 2

Comparison of the reconstructed network of *X. fastidiosa* CFBP 8418 with a draft network of *X. fastidiosa* Temecula1.

Reaction ID	Reaction name	Reaction formula	EC number	Pathway	Gene
Reactions absent in Temecula1 strain but identified in CFBP 8418					
R_DHPS2	Dihydropteroate synthase	1 M_4abz_c + 1 M_6hmhptpp_c -> 1 M_dhpt_c + 1 M_ppi_c	2.5.1.15	Folate biosynthesis and metabolism	(XFCFBP8418_014930)
R_ADCL	4-aminobenzoate synthase	1 M_4adcho_c <-> 1 M_pyr_c + 1 M_4abz_c + 1 M_h_c	4.1.3.38	Folate biosynthesis and metabolism	(XFCFBP8418_014810)
R_HSTPTr	Histidinol phosphate transaminase	1 M_imacp_c + 1 M_glu_L_c <-> 1 M_akg_c + 1 M_hisp_c	2.6.1.9	His metabolism	(XFCFBP8418_020310)
R_5DGLCNR	5-dehydro-D-gluconate reductase	1 M_5dglcn_c + 1 M_h_c + 1 M_nadph_c <-> 1 M_nadp_c + 1 M_glcn_c	1.1.1.69	Alternate carbon metabolism	(XFCFBP8418_010080)
Reactions absent in CFBP 8418 strain but identified in Temecula1					
R_POAACR	Peroxyaminoacrylate reductase	1 M_nadh_c + 1 M_poaac_c -> 1 M_3amac_c + 1 M_h2o_c + 1 M_nad_c	NA	Nucleotide Salvage Pathway	(WP_010892886.1)
R_EXOGLAS	Exoglucanase	1 M_14bglucan_e + 5 M_h2o_e -> 6 M_cellb_e	3.2.1.91	Plant cell wall degradation	(WP_010893773.1)
