# Supplementary material for: Genome-Scale Investigation of the Metabolic Determinants Generating Bacterial Fastidious Growth
Source: mSystems. 2020 Mar 31;5(2):e00698-19. doi: 10.1128/mSystems.00698-19 (PMC7112962; doi:10.1128/mSystems.00698-19)
Supplement: TEXT S4 [file mSystems.00698-19-s0004.pdf]

Mathematical modeling of metabolism

1. Quasi-steady state assumption [1]

A mass balance on the cell metabolism implies the formulation of equation 1.

$$\frac{dM}{dt} = K \cdot v \cdot B$$
$$v_i \geq 0 \; if \; v_i \; irreversible, i \in \{1; \ldots; r\} \quad (1)$$

M: metabolites concentrations vector
K: stoichiometric matrix of the metabolite network (size m × r)
v: reactions fluxes vector
B: biomass
Usual units are mM for M, $mM \cdot h^{-1} \cdot g_{dry\ weight}$ for v and $g_{dry\ weight}$ for B.

Biomass is defined in equation 2. In a metabolic network, it will be artificially represented by a biomass equation. [2]

$$B = M_{w,biomass\ metabolites} \cdot [\text{Biomass metabolites}] \quad (2)$$

$M_{w,biomass\ metabolites}$: molecular weight of metabolites constituting biomass
[Biomass metabolites]: metabolites constituting biomass
Metabolites constituting biomass are amino acids, cofactors, lipids and nucleic acids.

Metabolites can be separated into external metabolites consisting of substrates and products, internal metabolites and biomass. (eq. 3)

$$\frac{d\begin{pmatrix} S \\ C \\ P \\ B \end{pmatrix}}{dt} = \begin{pmatrix} K_S \\ K_C \\ K_P \\ K_B \end{pmatrix} \cdot v \cdot B$$
$$v_i \geq 0 \; if \; v_i \; irreversible, i \in \{1; \ldots; r\} \quad (3)$$

v: reaction flux vector
B: biomass
S: extracellular substrate concentration vector
C: internal metabolite concentration vector
P: excreted product concentration vector
K_S, K_C, K_P, K_B: subset of the complete stoichiometric matrix K corresponding to, respectively, extracellular substrates S, internal metabolites C, excreted products P and biomass B

Since the mathematical expression of v and the kinetics parameters related are often not known due to lack of experimental data, a quasi-stationary-state approximation (QSSA) is assumed: the metabolic concentration of internal metabolites are assumed constant (dC/dt=0). Biologically speaking, internal metabolites are assumed to be consumed immediately as they are produced. Only external metabolites and macromolecules constituting biomass such as membrane lipids and proteins are assumed to have slower dynamics leading to accumulation. This approximation is usually verified at the exponential phase of a bacterial growth. It allows to obtain the following system of equations (eq. 4).

$$\begin{cases} \dfrac{d\begin{pmatrix} S \\ P \\ B \end{pmatrix}}{dt} = \begin{pmatrix} K_S \\ K_P \\ K_B \end{pmatrix} \cdot v \cdot B \\ \dfrac{dC}{dt} = K_c \cdot v \cdot B \end{cases} \quad v_i \geq 0 \; if \; v_i \; irreversible, \; i \in \{1; \dots ; r\} \qquad (3')$$

$$\xRightarrow[\frac{dC}{dt}=0]{} \begin{cases} \dfrac{d\begin{pmatrix} S \\ P \\ B \end{pmatrix}}{dt} = \begin{pmatrix} K_S \\ K_P \\ K_B \end{pmatrix} \cdot v \cdot B \\ 0 = K_C \cdot v \end{cases} \quad v_i \geq 0 \; if \; v_i \; irreversible, \; i \in \{1; \dots ; r\} \qquad (4)$$

2. Flux Balance Analysis [1]

The metabolic fluxes are now the solution v of the system $\mathbf{K_c.}\cdot\mathbf{v = 0}$. However, this system is an under-determined system: in realistic metabolic model, there are more reactions than compounds (r>m), which leads to an infinity of solutions v. To reach a point within the solution space, an objective function **Z=f(v)** must be maximized or minimized. Generally, the objective function is assumed linear of the form $\mathbf{Z=c^Tv}$ with c the vector of weights, representing the contribution of each reaction to the objective function. An optimization solver will then be used to determine the metabolic fluxes upon a minimization or maximization objective function. This resolution of the system is called **Flux Balance Analysis (FBA)**.

Different scenarios of objective function can be used depending on the biological question investigated. Frequently, it is considered that microorganisms evolved by maximizing their growth rates (v_B), so the hypothesis that metabolic fluxes are shaped by growth maximization is reasonable. The scenario 1 is then widely used. In this scenario, a constant value is given to the substrate uptake flux (v_S) to prevent an unlimited biomass production. This value could be determined experimentally or approximated from literature. It is also possible to put as objective the minimization of the uptake rate and give a constant value (experimentally determined) for biomass growth (scenario 2).

Generally, FBA method gives a biomass flux superior than the experimental one, because there are unintegrated reactions such as housekeeping functions. An artificial ATP maintenance reaction is usually added in a metabolic network to unify fluxes between experimental and modeling data. ATP maintenance reaction consists in ATP hydrolysis: ATP → ADP + P. The flux value of this will be specified in the optimization problem.

max v_B

$$Kc \cdot v = 0$$
$$v_i \geq 0 \text{ if } v_i \text{ irreversible}$$
$$i \in \{1; \ldots; r\}$$
$$v_S = constant$$

Scenario 1, FBA

min v_S

$$Kc \cdot v = 0$$
$$v_i \geq 0 \text{ if } v_i \text{ irreversible}$$
$$i \in \{1; \ldots; r\}$$
$$v_B = constant$$

Scenario 2, FBA

The resolution of the system gives metabolic fluxes satisfying a maximization of biomass synthesis (or minimization of substrate uptake). However, some fluxes can take several values to reach this optimal biomass synthesis, which are not always biologically relevant. This is particularly the case in the presence of cycles in the metabolic network. Another formulation of FBA can then be formulated to find the minimal fluxes sustaining a maximal biomass flux (or minimal substrate uptake), with the first objective value integrated as a new constraint in a second optimization problem. An example is given in scenarios 3 and 4.

max v_B

$$Kc \cdot v = 0$$
$$v_i \geq 0 \text{ if } v_i \text{ irreversible}$$
$$i \in \{1; \ldots; r\}$$
$$v_S = constant$$

min $\sum_i |v_i|$

$$Kc \cdot v = 0$$
$$v_i \geq 0 \text{ if } v_i \text{ irreversible}$$
$$i \in \{1; \ldots; r\}$$
$$v_S = constant$$
$$v_B = v_{B,solved}$$

Scenario 3, FBA

min v_S

$$Kc \cdot v = 0$$
$$v_i \geq 0 \text{ if } v_i \text{ irreversible}$$
$$i \in \{1; \ldots; r\}$$
$$v_B = constant$$

min $\sum_i |v_i|$

$$Kc \cdot v = 0$$
$$v_i \geq 0 \text{ if } v_i \text{ irreversible}$$
$$i \in \{1; \ldots; r\}$$
$$v_S = constant$$
$$v_S = v_{S,solved}$$

Scenario 4, FBA

3. **Flux Variability Analysis** [3]

A drawback of FBA is that sometimes, several solutions v can satisfy the objective function. **Flux Variability Analysis (FVA)** allows finding the minimal and maximal flux that can carry each reaction while satisfying the optimal objective value found in FBA (e.g. maximal biomass flux or minimal substrate uptake rate).

The value determined through FBA is used as a new constraint, and FBA is computed for each metabolic flux, a first time by maximizing the metabolic flux and the second time by minimizing it.

To relax the system and enlarge the possibilities of fluxes variations, a deviation from the optimal flux can also be authorized: flux = optimal flux ± ε optimal flux (e.g ε = 1%). FVA scenario is presented below.

min or max v_k

$$Kc \cdot v = 0$$

$$v_i \geq 0 \text{ if } v_i \text{ irreversible}$$

$$i \in \{1; \dots; r\}$$

$$v_S = constant$$

for i in 1 to r

min / max v_i

$$Kc \cdot v = 0$$

$$v_i \geq 0, i \in \{1; \dots; r\}$$

$$v_s = constant$$

$$v_{k,solved} - \varepsilon v_{k,solved} < v_k < v_{k,solved} + \varepsilon v_{k,solved}$$

FVA

4. **Gene Deletion Study** [4]

In genome-scale metabolic networks, metabolic reactions are usually associated with the enzymatic complex catalyzing the reaction. It is then possible to determine if a gene is essential (*in silico*) to sustain growth or perform any other metabolic function. The algorithm to perform this essay is the following:

For each gene in the metabolic network:

- It is determined for which reactions the gene is necessary.

In the example below, the gene A is necessary for the metabolic reaction R1 but not for R2.

Reaction	Enzymatic complex
R1	(Gene A and Gene B) or (Gene A and Gene C)
R2	(Gene A) or (Gene D)

- For each reaction the gene is necessary, the flux of the reaction is set to zero.

On the previous example, the flux for R1 will be constrained to zero.

- FBA is then, performed with, for example, biomass maximization as objective. If no solution is obtained, then the gene is essential (*in silico*) to sustain growth. Otherwise the gene is facultative.

Similarly, the essentiality of reactions can be assessed by performing FBA with setting one by one metabolic fluxes to zero.

5. ***In silico*** **comparison of carbon substrates**

FBA can be alternatively performed on a range of identified carbon substrates. To compare rigorously the maximal growth rate generated by a given substrate, the uptake rates must be normalized by their number of carbon par molecule. For example, if we put 20 mmol·g_{DW}^{-1}·h^{-1} for glutamine (5 carbons), 16.67 mmol·g_{DW}^{-1}·h^{-1} will be put for glucose (6 carbon), to have the same carbon uptake rate (100 mmol C·g_{DW}^{-1}.h^{-1}).

The relative growth rates can be compared by computing the ratio:

$$Relative\ growth\ rate_{substrate\ i}\ (\%) = \frac{v_{biomass,substrate\ i}}{v_{biomass,substrate\ with\ highest\ biomass\ flux}} \times 100$$

6. Efficiency analysis

The efficiency of all the reactions available in a metabolic network to produce a molecule/macromolecule of interest, or biomass, can be analyzed using FBA.

The maximization of the molecule/macromolecule of interest production flux, or biomass flux, is put as objective. Additional constraints in the model (as ATP maintenance) are removed.

FBA solution fluxes include the CO_2 excretion, which represent the carbon losses from metabolic reactions.

The percentage of carbon loss upon the production, and subsequently the yield of production, can be determined:

$$Carbon\ loss\ (\%) = \frac{v_{CO_2,excreted}}{v_{substrate\ uptake} \times Number\ of\ carbon\ in\ substrate} \times 100$$

$$Yield\ of\ production\ (\%) = 100 - Carbon\ loss\ (\%)$$

References

1. Orth JD, Thiele I, Palsson BØ. What is flux balance analysis? Nat Biotechnol. 2010 Mar 1;28(3):245–8.
2. Feist AM, Palsson BO. The biomass objective function. Curr Opin Microbiol. 2010;13(3):344–9.
3. Mahadevan R, Schilling CH. The effects of alternate optimal solutions in constraint-based genome-scale metabolic models. Metab Eng. 2003 Oct 1;5(4):264–76.
4. Segrè D, Vitkup D, Church GM. Analysis of optimality in natural and perturbed metabolic networks. Proc Natl Acad Sci U S A. 2002 Nov 12;99(23):15112–7.

FlexFlux user guide

Guide to perform the simulations presented in
"Gerlin et al., *Genome-scale investigation of the metabolic determinants generating bacterial fastidious growth*"

If you want more information about the mathematical systems used in metabolic modeling (FBA, FVA, efficiency…) please consult the supplementary file "Mathematical modeling of metabolism".

1. Installing CPLEX.

The optimization software package IBM ILOG CPLEX Optimization Studio is used to solve linear programming problems in the study.

The software is free for students and academics upon registration to the IBM website, and can be easily downloaded there: https://my15.digitalexperience.ibm.com/b73a5759-c6a6-4033-ab6b-d9d4f9a6d65b/dxsites/151914d1-03d2-48fe-97d9-d21166848e65/technology/data-science.

2. Downloading and installing FlexFlux

FlexFlux is an open-source tool for metabolic flux and regulatory analysis, which is described there: http://lipm-bioinfo.toulouse.inra.fr/flexflux/documentation.html.

The webpage http://lipm-bioinfo.toulouse.inra.fr/flexflux/installation.html explains all the steps to download and install FlexFlux on Linux or Windows.
Briefly:

- Java 7 or higher must be installed on the computer
  http://www.oracle.com/technetwork/java/javase/downloads/index.html
- FlexFlux executable must be downloaded here:
  http://lipm-bioinfo.toulouse.inra.fr/flexflux/download/FlexFlux2.2.1.zip
- On Windows, FlexFlux is installed by unzipping the downloaded file in the folder of your choice.
- On Linux, you must have previously installed a solver, and then, you just need to run the commands from the folder where you put FlexFlux/.
  ```
  chmod +x Flexflux.sh
  chmod +x GraphicalFlexflux.sh
  ```

- The procedure to link CPLEX with FlexFlux is explained here:
  http://lipm-bioinfo.toulouse.inra.fr/flexflux/installation.html

3. Using FlexFlux

To perform metabolic modeling simulations (FBA, FVA, Gene Deletion Study…) using FlexFlux, you only need to:

1. Have the **SBML file of your metabolic network**
2. Have the **constraint file** specifying the constraints and the optimization problem to solve
3. Type a command line.

Alternatively, the graphical version of FlexFlux can be used using the command line GraphicalFlexflux.sh (Linux) or GraphicalFlexflux (Windows).

3.1. Performing FBA with substrate uptake minimization

To perform FBA on *X. fastidiosa*, you need the file glnConstraints.tab given in the folder flexflux/FBA/xfas in the github repository https://github.com/lgerlin/xfas-metabolic-model. The file specifies the two objectives functions: first, the minimization of glutamine (the carbon substrate) uptake and then minimization of the fluxes sum. All the other carbon substrates uptake apart from glutamine are constrained to 0, and ATP maintenance (R_ATPM), EPS production (R_DM_EPS_XF_e), virulence protein production (R_DM_LesA_e), biomass (R_DM_BIOMASS_c) are put to values determined by literature (see our Supplementary File 7 "Constraints used on metabolic modeling of *Xylella fastidiosa*").

The SBML file is given in the same folder.

To perform FBA, you need to place the constraints and SBML files in the same folder than the FlexFlux folder, and then use the command line:

- On Linux

```
Flexflux.sh FBA -cons glnConstraints.tab -out FBA_xfas_gln.txt -s XF_network.xml -sol CPLEX -ext -plot
```

- On Windows

```
Flexflux FBA -cons glnConstraints.tab -out FBA_xfas_gln.txt -s XF_network.xml -sol CPLEX -ext -plot
```

Alternatively, you can execute the command from the folder were the SBML and constraints file are located, and replace Flexflux or Flexflux.sh by /path/Flexflux or /path/Flexflux.sh, with /path/ the complete path to the Flexflux folder. Inversely, you can execute the command from the folder where Flexflux is located, and replace glnConstraints.tab and XF_network.xml by /path/glnConstraints.tab and /path/XF_network.xml, with /path/ the complete path to the Flexflux folder.

The result of the simulation (flux values) will be displayed graphically (as the argument "-plot" is activated in the command line). It will also be given in the file FBA_xfas_gln.txt. The objective value given is the sum of fluxes. The value of the glutamine uptake flux can be found at R_EX_gln_L_e line.

Similarly, FBA can be performed on the other metabolic network *R. solanacearum* with the constraint file available in flexflux/FBA/rsol (constraint values were put according to *R. solanacearum* metabolic model study by Peyraud et al., 2016) and the following command line:

- On Linux

```
Flexflux.sh FBA -cons glnConstraints.tab -out FBA_rsol_gln.txt -s rsolGMI1000_20190128_forModelling.xml -sol CPLEX -ext -plot
```

- On Windows

```
Flexflux.sh FBA -cons glnConstraints.tab -out FBA_rsol_gln.txt -s rsolGMI1000_20190128_forModelling.xml -sol CPLEX -ext -plot
```

3.2. Performing FBA to compare substrates

In the study, biomass production rates are compared for all *X. fastidiosa* carbon sources identified and being modeled.

Constraint files were generated for each substrate on flexflux/FBA/xfas_alternative-substrates. The objective function is now biomass maximization, and a value of substrate uptake was given. The value is normalized to provide the same value of carbon uptake (100 mmol $C \cdot h^{-1} \cdot g_{dry\ weight}^{-1}$). The minimization of the sum of fluxes was removed as only the biomass flux is studied there.

A similar command than the previous one can be used. glnConstraints.tab must be replaced by the appropriate substrate file (e.g glcConstraints.tab to study glucose) in the command line and the output file must also be renamed depending on the substrate (e.g FBA_xfas_glc.txt to study glucose).

For example, for glucose:

- On Linux

```
Flexflux.sh FBA -cons glcConstraints.tab -out FBA_xfas_glc.txt -s XF_network.xml -sol CPLEX -ext -plot
```

- On Windows

```
Flexflux FBA -cons glcConstraints.tab -out FBA_xfas_glc.txt -s XF_network.xml -sol CPLEX -ext -plot
```

3.3. Performing FBA to determine network efficiency (metabolic yields)

To determine the metabolic losses associated with biomass production on *X. fastidiosa*, you need the file biomassConstraints.tab given in the folder flexflux/FBA/xfas_efficiency-analysis in the github repository https://github.com/lgerlin/xfas-metabolic-model. The SBML file is also provided in the same folder.

The following command line is used:

- On Linux

```
Flexflux.sh FBA -cons biomassConstraints.tab -out FBA_xfas_biomass_efficiency.txt -s XF_network.xml -sol CPLEX -ext -plot
```

- On Windows

```
Flexflux FBA -cons biomassConstraints.tab -out FBA_xfas_biomass_efficiency.txt -s XF_network.xml -sol CPLEX -ext -plot
```

Similarly, metabolic losses associated with EPS or virulence protein can be determined. biomassConstraints.tab must be replaced by epsConstraints.tab or proteinConstraints.tab, and FBA_xfas_biomass_efficiency.txt must be replaced by FBA_xfas_eps_efficiency.txt or FBA_xfas_protein_efficiency.txt.

The same simulations can be performed on *R. solanacearum*, with the same command lines. The constraint and network files can be found in the folder flexflux/FBA/rsol_efficiency-analysis in the github repository https://github.com/lgerlin/xfas-metabolic-model. In the command lines, XF_network.xml must be replaced by rsolGMI1000_20190128_forModelling.xml.

3.4. Performing FBA to study the impact of FBPase activity on growth

The impact of the absence of inefficient FBPase is examined in the study. To compare optimal and suboptimal FBPase flux, constraint and SBML files are provided in flexflux/FBA/xfas_fbpase.

Considering an optimal FBPase, the following command line is used:

- On Linux

```
Flexflux.sh FBA -cons fbpoptConstraints.tab -out FBA_xfas_gln_fbpopt.txt -s XF_network.xml -sol CPLEX -ext -plot
```

- On Windows

```
Flexflux FBA -cons fbpoptConstraints.tab -out FBA_xfas_gln_fbpopt.txt -s XF_network.xml -sol CPLEX -ext -plot
```

Considering a suboptimal FBPase, the following command line is used:

- On Linux

```
Flexflux.sh FBA -cons fbpsuboptConstraints.tab -out FBA_xfas_gln_fbpsubopt.txt -s XF_network.xml -sol CPLEX -ext -plot
```

- On Windows

```
Flexflux FBA -cons fbpsuboptConstraints.tab -out FBA_xfas_gln_fbpsubopt.txt -s XF_network.xml -sol CPLEX -ext –plot
```

3.5. Performing FVA

To perform FVA on *X. fastidiosa*, you need the file glnConstraints.tab given in the folder flexflux/FVA/xfas in the github repository https://github.com/lgerlin/xfas-metabolic-model. The constraints are similar to the previous files. To have an appropriate comparison with other bacteria, constraints related to virulence (protein and EPS production) were removed. The ATP maintenance and glutamine uptake values chosen are identical to the ones determined experimentally for *R. solanacearum*. The objective function consists in maximizing biomass.

The SBML file is given in the same folder.

To perform FVA, you need to place the constraints and SBML files in the same folder than the FlexFlux folder, and then use the command line:

- On Linux

```
Flexflux.sh FVA -cons glnConstraints.tab -out FVA_xfas_gln.txt -lib 1 -s XF_network.xml -sol CPLEX -ext -plot
```

- On Windows

```
Flexflux FVA -cons glnConstraints.tab -out FVA_xfas_gln.txt -lib 1 -s XF_network.xml -sol CPLEX -ext -plot
```

The argument -lib 1 authorizes a deviation of the optimality of 1%.

FVA can also be performed on *R. solanacearum* and *E. coli* with the files given in flexflux/FVA/rsol and flexflux/FVA/ecol, and the following command line:

For *R. solanacearum*

- On Linux

```
Flexflux.sh FVA -cons glnConstraints.tab -out FVA_rsol_gln.txt -lib 1 -s rsolGMI1000_20190128_forModelling.xml -sol CPLEX -ext -plot
```

- On Windows

```
Flexflux FVA -cons glnConstraints.tab -out FVA_rsol_gln.txt -lib 1 -s rsolGMI1000_20190128_forModelling.xml -sol CPLEX -ext -plot
```

For *E. coli*

- On Linux

```
Flexflux.sh FVA -cons constraints.txt -out FVA_ecol.txt -lib 1 -s ecoli-orth2011.xml -sol CPLEX -ext -plot
```

- On Windows

```
Flexflux FVA -cons constraints.txt -out FVA_ecol.txt -lib 1 -s ecoli-orth2011.xml -sol CPLEX -ext –plot
```

3.6. Performing Gene Deletion Study (KO)

Gene Deletion Study or KO (knock-out) is performed in two different environmental conditions: glucose medium or protein-rich medium. The method is the same, only the constraint file is changed.

To perform Gene Deletion Study on *X. fastidiosa*, you need the file constraints.tab given in the folder flexflux/KO/xfas_glc in the github repository https://github.com/lgerlin/xfas-metabolic-model. The constraints are similar to the previous files. To have an appropriate comparison with other bacteria, constraints related to virulence (protein and EPS production) were removed. The ATP maintenance is identical to the one used for *R. solanacearum*, converted in this appropriate unit (h^{-1} replaced by day^{-1}). Glucose uptake values for *R. solanacearum* and *X. fastidiosa* were assumed to be proportional (using the number of mol of carbon) to the uptake rate for glutamine previously determined for the two organisms. The objective function consists in maximizing biomass.

The SBML file is given in the same folder.

To perform Gene Deletion, you need to place the constraints and SBML files in the same folder than the FlexFlux folder, and then use the command line:

- On Linux

```
Flexflux.sh KO -mode 1 -cons constraints.tab -out KO_genes.txt -s XF_network.xml -sol CPLEX -ext -plot
```

- On Windows

```
Flexflux KO -mode 1 -cons constraints.tab -out KO_genes.txt -s XF_network.xml -sol CPLEX -ext -plot
```

Reaction knock-out can also be performed: -mode 1 must be replaced by –mode 0, and the output file name must be replaced by KO_reactions.txt.

To test Gene Deletion Study and reaction knock-out on protein-rich environment, the constraints.tab file must be replaced by the file in the folder flexflux/KO/xfas_protein-rich.

Gene Deletion Study and reaction knock-out can also be performed on ***R. solanacearum***. The command lines are similar and the appropriate constraints are available in flexflux/KO/rsol_glc or flexflux/KO/rsol_protein-rich. For Gene Deletion Study:

- On Linux

```
Flexflux.sh KO -mode 1 -cons constraints.tab -out KO_genes.txt -s rsolGMI1000_20190128_forModelling.xml -sol CPLEX -ext –plot
```

- On Windows

```
Flexflux KO -mode 1 -cons constraints.tab -out KO_genes.txt -s rsolGMI1000_20190128_forModelling.xml -sol CPLEX -ext –plot
```

Similarly, reaction knock-out can also be performed: -mode 1 must be replaced by –mode 0, and the output file name must be replaced by KO_reactions.txt.

The simulations can also be performed on ***E. coli*** in the two environments, with the constraints and SBML files available at flexflux/KO/ecol_glc or flexflux/KO/ecol_protein-rich. For Gene Deletion Study, the command lines are:

- On Linux

```
Flexflux.sh KO -mode 1 -cons constraints.txt -out KO_genes.txt -s ecoli-orth2011.xml -sol CPLEX -ext –plot
```

- On Windows

```
Flexflux KO -mode 1 -cons constraints.txt -out KO_genes.txt -s ecoli-orth2011.xml -sol CPLEX -ext -plot
```

Similarly, reaction knock-out can also be performed: -mode 1 must be replaced by –mode 0, and the output file name must be replaced by KO_reactions.txt.
